# Beyond Tunnel Length: Tunnel Width–Derived Cover Index Improves Device Sizing and Predicts Outcomes After Transcatheter Patent Foramen Ovale Closure

**DOI:** 10.1016/j.jscai.2026.105391

**Published:** 2026-05-26

**Authors:** Tanuka D. Piech, Nicholas Ruggiero, Andrew C. Peters, Ashley Pender, Rebecca Marcantuono, Alec Vishnevsky, Praveen Mehrotra

**Affiliations:** a Division of Cardiology, Thomas Jefferson University Hospital, Thomas Jefferson University, Sidney Kimmel Medical College, Philadelphia, Pennsylvania; b Department of Cardiovascular Medicine, University of Michigan, Ann Arbor, Michigan

**Keywords:** cover index, patent foramen ovale, transcatheter closure, transesophageal echocardiography, tunnel width

## Abstract

**Background:**

Residual shunting after transcatheter patent foramen ovale (PFO) closure occurs in up to 25% of patients and increases the risk of recurrent neurologic events. Existing sizing strategies may be insufficient as they overlook the tunnel’s complex 3-dimensional (3D) anatomy. The aim of this study was to evaluate PFO tunnel dimensions with 3D transesophageal echocardiography (TEE) and determine whether tunnel width (TW) metrics would predict procedural outcomes.

**Methods:**

We retrospectively analyzed 102 patients undergoing PFO closure (n = 82 with follow-up bubble study). 3D-TEE was used to assess TW at right (TW_RA_) and left (TW_LA_) atrial openings, tunnel length, tunnel height at RA opening, and presence of septal aneurysm. We calculated an absolute TW_RA_ cover index for each patient as follows: RA disc diameter − TW_RA_. Receiver operating characteristic curve analysis was used to determine predictors of procedural outcomes.

**Results:**

Tunnel length did not correlate with TW_RA_, TW_LA_, or tunnel height; TW_RA_ (and not tunnel length) was significantly larger in patients with a septal aneurysm. TW_RA_ (area under the curve [AUC], 0.79; *P* < .001) and derived cover index (AUC, 0.88; *P* <.001) predicted residual shunting; a cover index of ≤9.5 mm provided optimal sensitivity (90%) and specificity (78%). The rate of residual shunting was 2% in patients with a cover index of >9.5 mm (median follow-up, 6.3 months). The TW_RA_ cover index also predicted the need for device upsizing (AUC, 0.99; *P* <.001; optimal cutoff ≤4.5 mm). Tunnel length did not predict either outcome.

**Conclusions:**

Despite its widespread use for sizing, tunnel length is unrelated to TW, septal excursion, or procedural outcomes after PFO closure. In contrast, TW-based metrics reliably predict outcomes, underscoring their potential to refine sizing strategies in patients undergoing this procedure.

## Introduction

Device sizing for transcatheter patent foramen ovale (PFO) closure remains highly variable in clinical practice.^1^ Although device manufacturers recommend using tunnel length, septal excursion, and septum secundum thickness for device size selection, these factors may not adequately describe the actual size of the PFO tunnel opening. Furthermore, with existing sizing techniques, residual shunting after transcatheter closure remains as high as 25%, which is associated with 3-fold increased risk of recurrent transient ischemic attack or stroke.^2^^,^^3^ Given these limitations, there is an increasing need for more precise, anatomy-specific approaches to tunnel characterization and device selection.

The PFO tunnel width (TW), which represents the major axis of the tunnel as measured by 3-dimensional (3D) transesophageal echocardiography (TEE), has been reported to be a better descriptor of PFO tunnel size.^4^, ^5^, ^6^ Although previous studies have described the importance of its assessment as early as 2010^7^^,^^8^ clinical adoption remains limited. The aims of this retrospective study were as follows: first, to describe the relationship between various PFO tunnel anatomic dimensions obtained with multiplanar 3D-TEE in patients referred for transcatheter closure; second, we hypothesized that that the TW (rather than the more commonly used tunnel length) and a derived cover index would predict long-term residual shunting and/or other procedural outcomes.

## Materials and methods

We retrospectively analyzed 102 patients who underwent transcatheter PFO closure at our institution between 2014 and 2025, who had either preprocedural or intraprocedural 3D-TEE imaging available. Eight patients underwent closure with 2-dimensional (2D) intracardiac echocardiography (ICE) guidance but had a preprocedural 3D-TEE study performed. Philips IE33 and/or General Electric E95 systems were used for preprocedural and/or intraprocedural 3D-TEE examinations. In patients undergoing 3D-TEE guidance, 3D postprocessing was performed using on-cart multiplanar reconstruction software, while offline analyses were performed with TomTec imaging software.

### PFO morphology assessment

**Figure 1:**
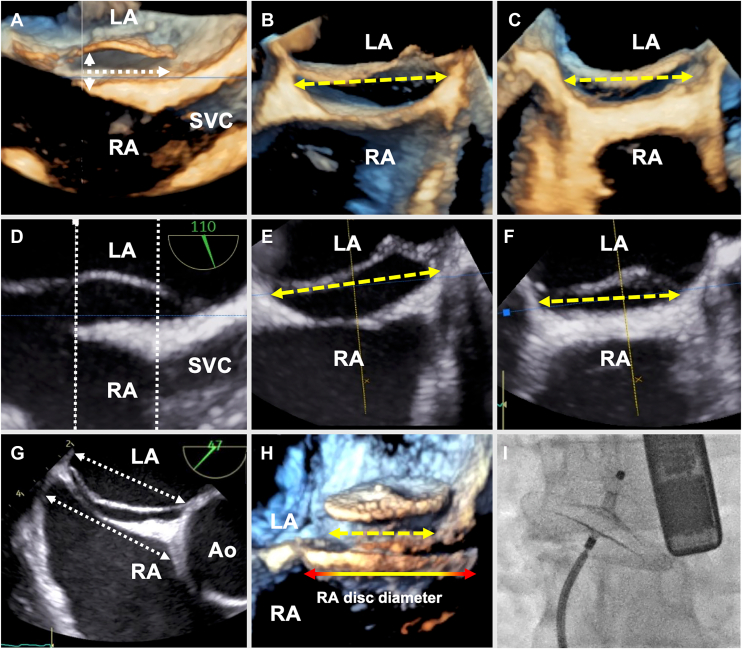
**Anatomic patent foramen ovale (PFO) dimensions and tunnel width (TW) sizing by 3D transesophageal echocardiography.** (**A**) 3D rendering of bicaval view at 110°, depicting tunnel length (horizontal dotted white arrow) and height (vertical dotted white arrow). En face 3D rendering of PFO TW_RA_ (**B**) and PFO TW_LA_ (**C**), with measurements shown with dashed yellow arrow. Bicaval 3D multiplanar image (**D**) at 110° of PFO tunnel, depicting transverse imaging planes (dotted white lines) used to create orthogonal multiplanar views for measurement of TW_RA_ (**E**) and TW_LA_ (**F**). (**G**) Midesophageal view at 47° depicting approximate atrial dimensions to ensure discs of closure device are adequately accommodated. 3D rendering (**H**) and fluoroscopic image (**I**) of PFO closure device. Ao, aorta; LA, left atrium; RA, right atrium; SVC, superior vena cava.

Anatomic PFO dimensions were measured retrospectively on preprocedural 3D-TEE images in the 8 ICE-guided cases and prospectively (except for tunnel height) in patients undergoing closure with 3D-TEE guidance (Figure 1). Patients undergoing closure without any 3D imaging were not included. Multiplanar 3D-TEE image acquisition of the PFO tunnel was performed in the bicaval view during maximum separation of the septum primum and secundum to facilitate accurate tunnel measurements. Single-beat zoom mode was used, with settings optimized for high spatial resolution and a minimum temporal resolution of 10 to 15 Hz.

The following dimensions were measured: TW at both right (TW_RA_) and left (TW_LA_) atrial openings, tunnel length, and tunnel height at right atrial (RA) opening. The TW is defined as the major axis of the oval-shaped PFO opening and is orthogonal to both the tunnel height and length.^4^ The tunnel length was measured as the distance between the RA opening at the fossa ovalis and the left atrial (LA) opening; care was taken not to overmeasure the tunnel length due to its crescentic opening in the left atrium. The tunnel height—the dynamic minor axis of the oval opening and a marker of septal excursion—was recorded as the maximal separation between the septum primum and septum secundum at the RA opening. The presence of atrial septal aneurysm (ASA) as defined by current guidelines^9^ was also documented for all patients.

### Device sizing and TW cover index

In all patients, device sizing adhered to manufacturer guidelines.^10^ However, in the 94 patients who underwent intraprocedural 3D-TEE guidance—where the operator had real-time knowledge of TW measurements—device sizing was also considered inappropriate by the operator if the RA disc diameter was smaller than the TW_RA_ primarily owing to concern for device instability or embolization. A minimum degree of TW oversizing was not prespecified early during our experience as the optimal extent of oversizing required was not known at that time.^4^ Anteroposterior atrial dimensions were also routinely assessed for larger sized devices to ensure discs could be adequately accommodated (Figure 1G).

We retrospectively determined an average absolute TW_RA_ cover index for each patient as follows:

$${\text{TW}}_{\text{RA}}\;\text{Cover}\;\text{Index}\;\left(\text{Absolute}\right)=\text{RA}\;\text{Disc}\;\text{Diameter}-{\text{TW}}_{\text{RA}}$$

A relative cover index was also calculated as follows:

$${\text{TW}}_{\text{RA}}\;\text{Cover}\;\text{Index}\;\left(\text{Relative}\right)=\frac{{\text{TW}}_{\text{RA}}\;\text{Cover}\;\text{Index}\;\left(\text{Aboslute}\right)}{\text{RA}\;\text{Disc}\;\text{Diameter}}$$

The TW_RA_ cover index concept is depicted in the Central Illustration.^4^

**Central Illustration:**
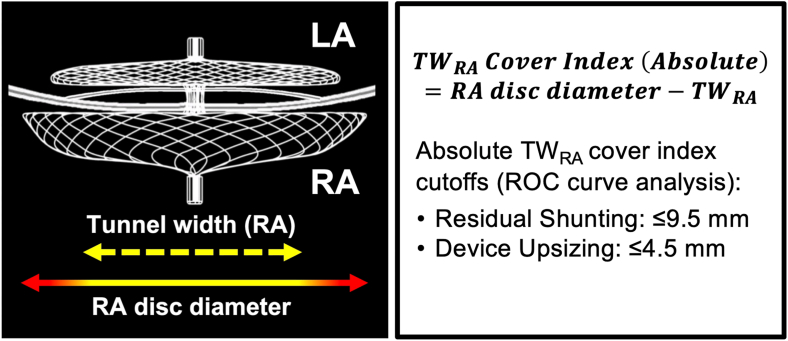
**Tunnel width (TW) cover index concept.** When the right atrial disc (multicolor arrow) exceeds the TW_RA_ (dashed yellow arrow), thrombi will not be able to pass through the tunnel along the margins of the device, nor will the device embolize into the left atrium (LA) through the tunnel (left). In receiver operating characteristic (ROC) curve analysis, the absolute TW_RA_ cover index strongly predicts residual shunting at follow-up and need for device upsizing (right). Reprinted with permission from Datta et al.^4^ RA, right atrium.

### End points

Study end points were assessed retrospectively. The primary end point was the presence of residual shunting at the time of long-term follow-up echocardiogram. Residual shunting was defined as a positive bubble study and graded by counting the number of microbubbles in the left ventricle after administration of agitated saline (within 3 cardiac cycles) as follows: grade 1 (small): 1 to 10 microbubbles; grade 2 (moderate): 11 to 30 microbubbles; and grade 3 (large): >30 microbubbles. Long-term follow-up bubble studies were available in 82 patients (n = 6 with ICE and n = 76 with 3D-TEE guidance). Secondary end points included need for device upsizing (due to device instability along interatrial septum during tug test or device embolization), new-onset atrial fibrillation, recurrent embolic events, and device erosion.

### Statistical analysis

Variables are expressed as mean ± SD or number (percentage). Pearson correlation coefficients were used to define the relationship between anatomic PFO dimensions by 3D-TEE. The Mann–Whitney *U* test was used to compare tunnel metrics between patients with and without an ASA as well as between those with and without residual shunting. The Kruskal–Wallis and χ^2^ tests were used to compare tunnel indices according to device size. Significance values for pairwise comparisons were adjusted using the Bonferroni correction for multiple tests. Interobserver and intraobserver variability for 3D-TEE based PFO measurements was assessed with intraclass correlation coefficients. Receiver operating characteristic (ROC) curve analysis was used to determine the ability of tunnel dimensions and cover indices to predict outcomes. The maximal value of Youden index was used to determine the optimal cutoff for PFO dimensions and cover indices in ROC curve analysis. SPSS software (version 29) was used for all statistical analyses.

## Results

Baseline characteristics are shown in Table 1, while baseline shunt and procedural characteristics are shown in Table 2. The majority of PFO closures were performed for cryptogenic stroke, while the most commonly used device was the Amplatzer 25-mm PFO occluder (Abbott Laboratories). The mean risk of paradoxical embolism score in our cohort was 6 ± 2.

**Table 1:** Baseline characteristics.

Characteristics	N = 102
Age, y	49 ± 12
Female sex	53 (52)
Weight, kg	85 ± 21
Height, m	1.7 ± 0.1
Comorbidities	
Hypertension	34 (33)
Hyperlipidemia	49 (48)
Coronary artery disease	12 (12)
Diabetes mellitus	10 (10)
Congestive heart failure	3 (3)
Obstructive sleep apnea	21 (21)
Migraine disorder	47 (46)
Implantable loop recorder	48 (47)
Tobacco use^a^	26 (25)
Preclosure medications^b^	
Aspirin	67 (67)
Clopidogrel	32 (31)
Statin	78 (77)
Warfarin	2 (2)
Direct oral anticoagulant	39 (38)

Data are expressed as mean ± SD or as n (%).

a Includes former and current tobacco use.

b Only preclosure medications are shown. The postclosure antithrombotic regimen uniformly consisted of indefinite aspirin and clopidogrel for 6 months, except for patients with an indication for lifelong anticoagulation. In these cases, clopidogrel was substituted for an anticoagulant, while aspirin was generally discontinued after 6 months. Continuation of statin therapy after closure was at the discretion of the patient’s cardiologist.

**Table 2:** Baseline shunt and procedural characteristics.

Characteristics	Total cohort (N = 102)	With follow-up bubble study (n = 82)
Baseline shunt characteristics^a^		
Grade 1	20 (20)	17 (22)
Grade 2	31 (31)	24 (30)
Grade 3	48 (49)	38 (48)
ASA	30 (29)	24 (29)
Indication for closure		
Cryptogenic stroke	95 (93)	77 (94)
TIA or PAE	5 (5)	4 (5)
POS	2 (2)	1 (1)
Occluder type^b^		
PFO	93 (91)	75 (91)
Cribriform	9 (9)	7 (9)
Device size^c^, mm		
25	78 (76)	62 (76)
30	16 (16)	13 (16)
35	8 (8)	7 (8)

Data are expressed as n (%).

ASA, atrial septal aneurysm; PAE, paradoxical arterial embolism, PFO, patent foramen ovale; POS, platypnea-orthodeoxia syndrome, TIA, transient ischemic attack.

a Baseline shunt severity was available only in 99 patients and obtained from the preclosure transthoracic echocardiography (TTE) or transesophageal echocardiography (when TTE not available), using the same criteria for grading postclosure residual shunt.

b All devices used were Amplatzer devices (Abbott Laboratories).

c Device size was defined by right atrial disc size.

### 3D-TEE PFO morphology analysis

**Figure 2:**
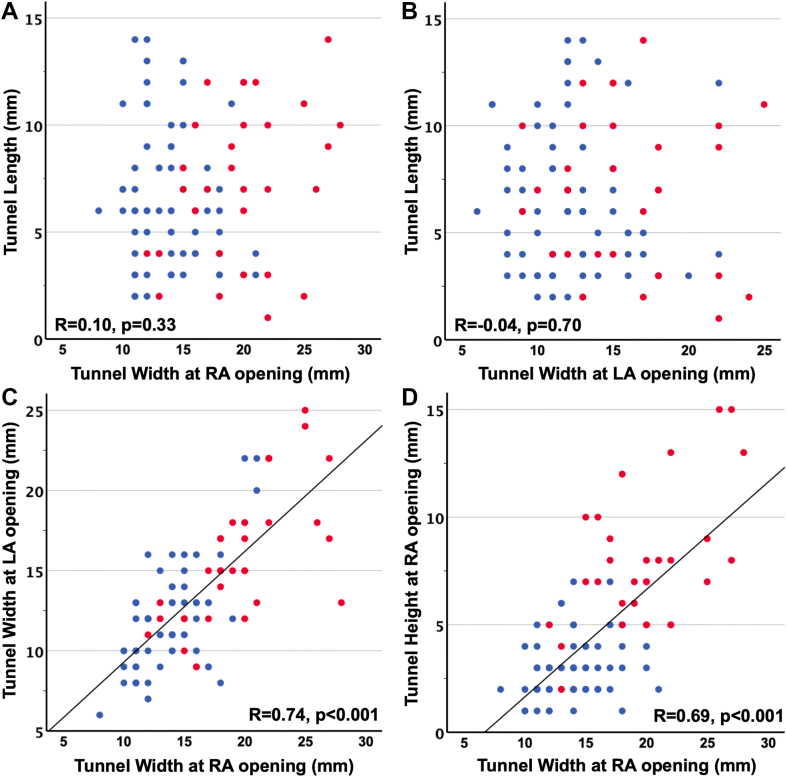
**Scatter diagrams of anatomic patent foramen ovale (PFO) dimensions by multiplanar 3D transesophageal echocardiography.** There was no correlation between tunnel length and tunnel width (TW) at the right atrium (TW_RA_; **A**) or at left atrium (TW_LA_; **B**). In contrast, there were strong linear relationships between TW_RA_ and TW_LA_ (**C**) and TW_RA_ and maximum tunnel height at right atrial opening (**D**). There was a modest correlation (*R* = 0.37; *P* < .001) between TW at the left atrial opening and tunnel height and no significant correlation (*R* = 0.19; *P* = .06) between tunnel height and length (plots not shown). Red and blue colors represent patients with and without atrial septal aneurysm, respectively.

**Supplemental Figure 1:**
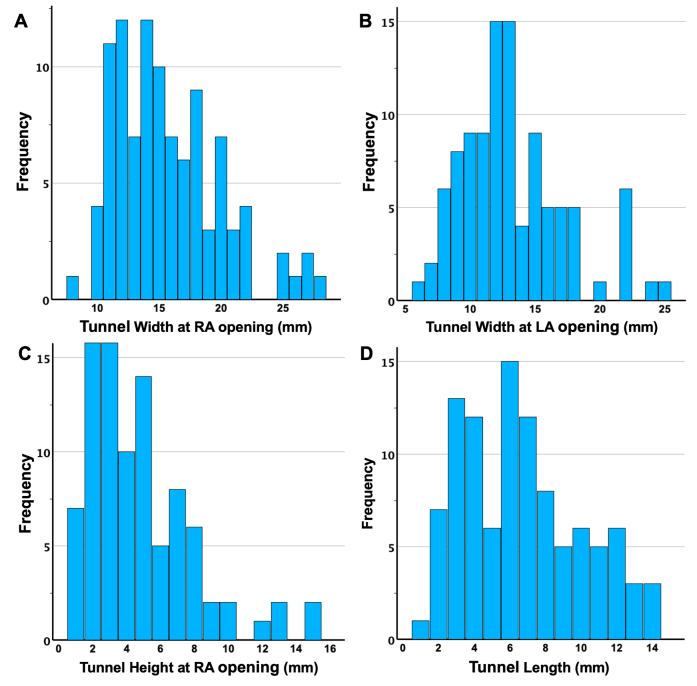
Distribution of anatomic PFO dimensions by 3D-TEE in patients referred for transcatheter PFO closure (n = 102).

By 3D-TEE multiplanar analysis, the average TW_RA_ was 16 ± 4 mm, TW_LA_ was 13 ± 4 mm, tunnel length was 7 ± 3 mm, and maximal tunnel height at the RA opening was 5 ± 3 mm. Histograms for all PFO dimensions are shown in Supplemental Figure S1. In most patients (73%, n = 74), the TW_RA_ exceeded the TW_LA_, while in 18% (n = 18), the widths were equal (mean difference, 3 mm; range, −4 to 15 mm). There was no correlation (Figure 2) between tunnel length and TW_RA_ (*R* = 0.10; *P* = .33), TW_LA_ (*R* = −0.04; *P* = .70), or tunnel height (*R* = 0.19; *P* = .06). In contrast, there were strong correlations between TW_RA_ and TW_LA_ (*R* = 0.74; *P* <.001) and between TW_RA_ and maximum tunnel height at RA opening (*R* = 0.69; *P* <.001). ASA was present in 30 (29%) patients. Differences in tunnel dimensions according to presence or absence of ASA are shown in Table 3. Interobserver and intraobserver variability in a sample of 12 patients was 0.9 and 0.94; 0.81 and 0.89; 0.93, and 0.91; and 0.96 and 0.89 for TW_RA_, TW_LA_, tunnel length, and tunnel height, respectively. Tunnel dimensions and cover indices according to device size are shown in Table 4.

**Table 3:** 3D-TEE PFO dimensions in patients with and without atrial septal aneurysm.

	Atrial septal aneurysm (n = 30)	No atrial septal aneurysm (n = 72)	*P*
TW_RA_, mm	20 ± 4	14 ± 3	<.001
TW_LA_, mm	16 ± 4	12 ± 3	<.001
Tunnel height at RA opening, mm	8 ± 3	3 ± 2	<.001
Tunnel length, mm	7 ± 4	7 ± 3	.45

Data are expressed as mean ± SD.

LA, left atrium; PFO, patent foramen ovale; RA, right atrium; TEE, transesophageal echocardiography; TW, tunnel width.

**Table 4:** Cover indices and PFO morphology according to device size.

	25 mm (n = 78)	30 mm (n = 16)	35 mm (n = 8)	*P*
TW_RA_, mm	14 ± 3^a^^,^^b^	20 ±3	24 ± 3	<.001
TW_LA_, mm	12 ± 3^a^^,^^b^	16 ± 4	21 ±3	<.001
Tunnel height at RA opening, mm	4 ± 2^a^^,^^b^	7 ± 3	9 ± 5	<.001
Tunnel length, mm	7 ± 3	6 ± 3	9 ± 3	.08
TW_RA_ cover index (absolute)	11 ± 3	10 ± 3	11 ± 3	.41
TW_RA_ cover index (relative), %	44 ± 11^a^^,^^b^	33 ± 9	32 ± 8	<.001
Atrial septal aneurysm	12 (15)^a^^,^^b^	12 (75)	6 (75)	<.001

Data are expressed as mean ± SD or as n (%).

LA, left atrium; RA, right atrium; PFO, patent foramen ovale; TW, tunnel width.

a *P* < .05 vs 30 mm.

b *P* < .05 vs 35 mm.

### Study end points

**Figure 3:**
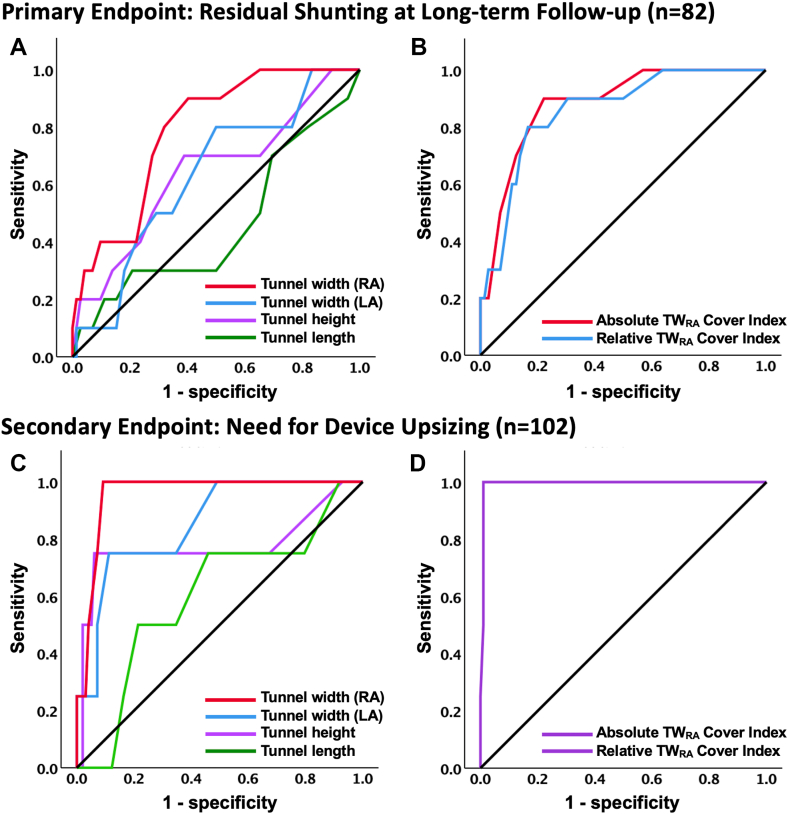
**Receiver operating characteristic (ROC) curve analysis.** ROC curve analysis was performed to evaluate the ability of anatomic PFO dimensions (left) and tunnel width (TW) cover indices (right) obtained with 3D transesophageal echocardiography to predict long-term residual shunting (**A** and **B**; n = 82) and need for device upsizing (**C** and **D**; n = 102). Optimal cutoff values for absolute TW at the right atrium (TW_RA_) cover index were ≤9.5 mm (sensitivity, 90%; specificity 78%) and ≤4.5 mm (sensitivity, 100%; specificity, 99%) for predicting residual shunting and device upsizing, respectively.

The primary end point of long-term residual shunting after closure was observed in 10 of the 82 (12%) patients (grade 1, n = 1; grade 2, n = 5; and grade 3, n = 4) on follow-up bubble study (median follow-up, 6.5 months). In ROC curve analysis (Table 5), TW_RA_ was the only PFO dimension that was predictive of residual shunting (area under the curve [AUC], 0.79; *P* <.001), with a cutoff of ≥15.5 mm having the best combination of sensitivity (89%) and specificity (65%) (Figure 3A).

**Table 5:** Receiver operating characteristic curve analysis.

Parameter	AUC	95% CI	*P*
Residual shunting at long-term follow-up (n = 82)			
TW_RA_ cover index (absolute)	0.88	0.78-0.98	<.001
TW_RA_ cover index (relative)	0.86	0.75-0.97	<.001
TW_RA_	0.79	0.66-0.92	<.001
Tunnel height at RA opening	0.64	0.45-0.84	.16
TW_LA_	0.63	0.46-0.81	.13
Tunnel length	0.46	0.25-0.67	.70
Need for device upsizing (n = 102)^a^			
TW_RA_ cover index (absolute)	0.99	0.98-1.00	<.001
TW_RA_ cover index (relative)	0.99	0.98-1.00	<.001
TW_RA_	0.96	0.91-1.00	<.001
TW_LA_	0.86	0.67-1.00	<.001
Tunnel height at RA opening	0.78	0.45-1.10	.10
Tunnel length	0.48	0.32-0.89	.70

AUC, area under curve; LA, left atrium; RA, right atrium; PFO, patent foramen ovale; TW, tunnel width.

a Cover indices for initial failed deployment (n = 4) were used in the receiver operating characteristic curve analysis for device upsizing end point.

Both absolute (AUC, 0.88; *P* <.001) and relative (AUC, 0.86; *P* <.001) TW_RA_ cover indices were strongly predictive of residual shunting at follow-up (Figure 3B). For the absolute TW_RA_ cover index, a cutoff of ≤9.5 mm had the best combination of sensitivity (90%) and specificity (78%), while for the relative TW_RA_ cover index, the optimal cutoff was ≤33% (sensitivity, 80%; specificity, 83%). When the cohort was restricted to patients with an absolute TW_RA_ cover index of >9.5 mm, the residual shunting rate decreased to 2% (1/57; median follow-up, 6.3 months). When limited to patients with a relative TW_RA_ cover index of >33%, the residual shunting rate was 3% (2/62; median follow-up, 6.5 months). A cover index derived from the TW_LA_ did not predict residual shunting. In patients without any long-term residual shunting (n = 72), the mean absolute and relative cover indices were 11 ± 2 mm and 43% ± 10%, respectively (Supplemental Table S1).

Device upsizing was needed in 4 patients (3 for device instability with initial deployment and 1 for device embolization^6^) after failed implantation of a 25-mm device. TW_RA_ (AUC, 0.96; optimal cutoff, ≥20.5 mm; sensitivity, 100%; specificity, 91%), TW_LA_ (AUC, 0.86; optimal cutoff, ≥17.5 mm; sensitivity, 75%; specificity, 89%), absolute TW_RA_ cover index (AUC, 0.99; optimal cutoff, ≤4.5 mm; sensitivity, 100%; specificity, 99%), and relative TW_RA_ cover index (AUC, 0.99; optimal cutoff, ≤16%; sensitivity, 100%; specificity, 99%) were strongly predictive (*P* < .001) of this end point (Table 5, Figure 3C, D). Tunnel length did not predict need for device upsizing. Of these 4 patients, 2 underwent closure with ICE guidance, with cover indices of 0 and −3 mm with the initial failed devices. The rate of atrial fibrillation after closure was 8% (8/102). There was no pericardial effusion, device erosion, or recurrent embolic events observed in any patients after closure.

## Discussion

The key findings of our study are as follows: (1) the PFO TW, which reflects the true size of the tunnel opening does not correlate with tunnel length despite the latter being recommended by device manufacturers and routinely used by interventionalists for device sizing during transcatheter PFO closure; (2) TW, and not tunnel length, is related to septal excursion as described by the maximal tunnel height at RA opening and presence of an ASA; (3) TW_RA_ and derived cover index (and not tunnel length) are strongly predictive of residual interatrial shunting at follow-up bubble study and need for device upsizing; and (4) a sizing strategy that incorporates TW metrics may enhance device selection and improve outcomes after PFO closure.

### Standardizing PFO tunnel nomenclature

The PFO is a complex 3D structure formed by the flap-like, thin septum primum and thicker septum secundum and most closely resembles an oval-shaped tunnel with RA and LA openings.^11^ Although the tunnel length is frequently used to describe the size of the PFO,^10^^,^^12^ this dimension merely represents the distance between the 2 openings rather than actual size of the opening itself.^11^ The tunnel height represents the dynamic separation between the septum primum and secundum and represents the minor axis of the oval opening,^13^^,^^14^ although the term TW has also been used to describe this dimension in the literature.^15^ We reserve the term TW to describe the major axis of the oval-shaped RA and LA openings.^4^ The TW exists in a plane orthogonal to the tunnel length and height and, unlike these latter dimensions, is best assessed with multiplanar 3D (or biplane) imaging since it is not easily visualized on standard 2D planes.

### Current sizing methods and cover index concept

Defect sizing for transcatheter PFO closure remains highly variable in clinical practice.^1^^,^^10^^,^^12^^,^^16^ Although some advocate for balloon sizing,^1^^,^^17^ most contemporary approaches rely on a combination of PFO tunnel length, septal excursion, and septum secundum thickness with a longer tunnel length prompting selection of a larger sized device.^10^ In our cohort, some patients with relatively smaller tunnel lengths ultimately required larger devices due to a markedly increased TW measurements. Notably, in 4 cases in our cohort, where the initial smaller device was selected based on the relatively smaller tunnel length—as recommended by the manufacturer—the device failed to seat properly necessitating upsizing. Importantly, even with the use of larger devices in these patients, no severe adverse events related to TW oversizing (eg, pericardial effusion or device erosion) were observed.

Although the concept of a cover index or device oversizing is well-established in other structural heart interventions, including transcatheter aortic valve implantation,^18^ LA appendage closure,^19^ and atrial septal defect closure,^11^ this study is the first to describe its application in transcatheter PFO closure. The theoretical rationale is that oversizing based on TW enhances device stability, reduces the risk of device embolization, and minimizes peridevice shunting. Because the PFO closure device tends to center at the tunnel’s RA opening, the TW_RA_—and not TW_LA_—is more optimally suited for determination of the cover index. Lastly, our findings support prior recommendations from other operators that patients with larger TWs require larger-sized devices to achieve adequate tunnel closure.^5^^,^^8^

### A new sizing paradigm for PFO closure

While transcatheter PFO closure is recommended by the current Society for Cardiovascular Angiography & Interventions (SCAI) guidelines^20^ to reduce the risk of recurrent stroke in patients with cryptogenic stroke, residual shunting after closure remains as high as 25% and is associated with an increased risk of recurrent stroke.^2^^,^^3^^,^^21^ The lower residual shunting rate observed in our study (12%) likely reflects our gradual increasing experience with the application of TW measurements within our sizing strategy. These observations highlight the need for a refined approach to device sizing for transcatheter PFO closure. First, the chosen device size should achieve TW_RA_ cover index of 9 to 10 mm (ensuring 4-5 mm RA disc coverage on either side of the RA tunnel opening) to minimize risk of peridevice right-to-left shunting. A TW_RA_ cover index of <5 mm should be avoided as it increases risk of device instability. While 9 to 10 mm of coverage by the RA disc should be safe in most patients, avoiding significant left-sided disc oversizing is an important consideration to ensure adequate positioning relative to the aorta and to avoid device erosion. If this of concern, evaluation for an alternative device type should be considered. Second, there remains value in fully covering the entire tunnel length as residual septum primum tissue on the LA side could serve as a blind pouch and nidus for thrombus formation. However, whether the tunnel length is fully covered is determined primarily by the size of the LA disc, which helps appose the thin septum primum to the thicker secundum tissue. Lastly, for larger devices, to reduce risk of device erosion, we routinely measure the anteroposterior LA and RA dimensions in a midesophageal 0° to 45° view to confirm safe accommodation of the discs without impingement on the posterior atrial wall or aorta. The presence of ASA and thick septum secundum may further support selection of a larger device in cases of borderline measurements, as currently recommended.^10^

Regardless of the modality chosen for intraprocedural guidance (TEE or ICE), our study highlights that reliance on tunnel length alone may be insufficient and that TW metrics (obtained either with preprocedural or intraprocedural 3D imaging) should be incorporated into existing sizing strategies. TW dimensions may also be obtainable using 3D-ICE; however, it remains uncertain whether a very large TW can be adequately visualized, given the technique’s relatively limited field of view. Notably, many patients with residual shunts in our study had very large TW dimensions and/or minimal device coverage (Supplemental Table S1). Finally, in cases where the TW is not readily discernible by TEE or ICE, passage of a guide wire through the tunnel can make this dimension more apparent by inducing mechanical separation of the septum primum from secundum (wire sizing).^4^, ^5^, ^6^

### Limitations and future directions

Our study is limited by its single-center, retrospective design and the modest size of our cohort. Despite these limitations, we observed a strong association between TW indices and study outcomes—a relationship that could be explored further in future device trials. Our study did not include a comparison group using 2D or 3D ICE-based sizing; however, this is a potential avenue for future study. Our center primarily uses devices made only by 1 manufacturer; however, the TW_RA_ cover index concept should also be applicable to other manufacturers’ closure devices. Obtaining accurate TW measurements with multiplanar 3D-TEE requires training but should be readily achievable by experienced structural echocardiographers, while approximate TW estimates can also be obtained with biplane imaging.^6^ Lastly, complete endothelialization of implanted closures device may not occur in some cases until 12 months after device implantation, and our findings may have differed if some follow-up bubble studies were performed later.

## Conclusions

Tunnel length does not correlate with the true size of the PFO’s tunnel opening (ie, the TW) or septal excursion. Moreover, it is the TW_RA_ and the derived cover index obtained with 3D-TEE—not the traditionally used tunnel length—that predicts long-term residual shunting and need for device upsizing during transcatheter PFO closure. Our findings challenge the sole reliance on tunnel length for device selection and support incorporation of TW-based metrics to optimize sizing strategies and improve procedural outcomes. Prospective validation of our findings is warranted.

## CRediT authorship contribution statement

**Tanuka D. Piech:** Conceptualization, Data curation, Formal analysis, Investigation, Methodology, Validation, Writing – original draft, Writing – review & editing. **Nicholas Ruggiero:** Conceptualization, Investigation, Methodology, Supervision, Writing – original draft, Writing – review & editing. **Andrew C. Peters:** Conceptualization, Data curation, Formal analysis, Investigation, Methodology, Supervision, Writing – original draft, Writing – review & editing. **Ashley Pender:** Conceptualization, Data curation, Investigation, Methodology, Writing – original draft, Writing – review & editing. **Rebecca Marcantuono:** Conceptualization, Data curation, Methodology, Supervision, Writing – review & editing. **Alec Vishnevsky:** Conceptualization, Data curation, Formal analysis, Investigation, Methodology, Supervision, Writing – original draft, Writing – review & editing. **Praveen Mehrotra:** Conceptualization, Data curation, Formal analysis, Investigation, Methodology, Supervision, Writing – original draft, Writing – review & editing.

## Supplementary material

Supplementray Table 1
