# Supplementary material for: Beyond Tunnel Length: Tunnel Width–Derived Cover Index Improves Device Sizing and Predicts Outcomes After Transcatheter Patent Foramen Ovale Closure
Source: J Soc Cardiovasc Angiogr Interv. 2026 May 26;5(7):105391. doi: 10.1016/j.jscai.2026.105391 (PMC13400113; doi:10.1016/j.jscai.2026.105391)
Supplement: Supplementray Table 1 [file mmc1.docx]

**Supplemental** **Table 1. Cover indices and PFO morphology according to presence or absence of residual shunt (n=82)**

|  | **No residual Shunt**  **(n=72)** | **Residual shunt present**  **(n=10)** | **P-value** |
| --- | --- | --- | --- |
| TW_RA_ (mm) | 15 ± 4 | 20 ± 5 | .003 |
| TW_LA_ | 13 ± 4 | 15 ± 4 | .17 |
| Tunnel height at RA opening | 4 ± 3 | 7 ± 5 | .15 |
| Tunnel length | 7 ± 3 | 7 ± 4 | .67 |
| TW_RA_ cover index (absolute) | 11 ± 2 | 7 ± 3 | <.001 |
| TW_RA_ cover index (relative) (%) | 43 ± 10 | 26 ± 13 | <.001 |
| Atrial Septal Aneurysm, *n* (%) | 19 (26) | 5 (50) | .12 |

Data are expressed as mean ± SD or as number (percentage).
